# Immune Imprinting Drives Human Norovirus Potential for Global Spread

**DOI:** 10.1128/mbio.01861-22

**Published:** 2022-09-14

**Authors:** Lisa C. Lindesmith, Florencia A. T. Boshier, Paul D. Brewer-Jensen, Sunando Roy, Veronica Costantini, Michael L. Mallory, Mark Zweigart, Samantha R. May, Helen Conrad, Kathleen M. O’Reilly, Daniel Kelly, Cristina C. Celma, Stuart Beard, Rachel Williams, Helena J. Tutill, Sylvia Becker Dreps, Filemón Bucardo, David J. Allen, Jan Vinjé, Richard A. Goldstein, Judith Breuer, Ralph S. Baric

**Affiliations:** a Department of Epidemiology, University of North Carolina, Chapel Hill, North Carolina, USA; b Department of Infection, Immunity and Inflammation, UCL Great Ormond Street Institute of Child Health, University College London, London, United Kingdom; c Division of Viral Diseases, Centers for Disease Control and Prevention, Atlanta, Georgia, USA; d Centre for Mathematical Modelling of Infectious Diseases, Department of Infectious Disease Epidemiology, London School of Hygiene and Tropical Medicine, London, United Kingdom; e Department of Infection Biology, Faculty of Infectious and Tropical Diseases, London School of Hygiene and Tropical Medicine, London, United Kingdom; f Enteric Virus Unit, The Virus Reference Department, UK Health Security Agency, London, United Kingdom; g Department of Genetics & Genomic Medicine, UCL Great Ormond Street Institute of Child Health, University College London, London, United Kingdom; h Department of Family Medicine, University of North Carolina at Chapel Hill, Chapel Hill, North Carolina, USA; i Department of Microbiology, National Autonomous University of Nicaragua, León, León, Nicaragua; j Division of Infection and Immunity, University College London, London, United Kingdom; k Department of Microbiology, Great Ormond Street Hospital for Children NHS Foundation Trust, London, United Kingdom; The Catholic University of America; Catholic University of America

**Keywords:** norovirus, neutralizing antibodies, surveillance, epidemic, histo-blood group antigens, antigenic cartography, variants of concern, antigenic seniority, immune imprinting, sequencing, variant persistence

## Abstract

Understanding the complex interactions between virus and host that drive new strain evolution is key to predicting the emergence potential of variants and informing vaccine development. Under our hypothesis, future dominant human norovirus GII.4 variants with critical antigenic properties that allow them to spread are currently circulating undetected, having diverged years earlier. Through large-scale sequencing of GII.4 surveillance samples, we identified two variants with extensive divergence within domains that mediate neutralizing antibody binding. Subsequent serological characterization of these strains using temporally resolved adult and child sera suggests that neither candidate could spread globally in adults with multiple GII.4 exposures, yet young children with minimal GII.4 exposure appear susceptible. Antigenic cartography of surveillance and outbreak sera indicates that continued population exposure to GII.4 Sydney 2012 and antigenically related variants over a 6-year period resulted in a broadening of immunity to heterogeneous GII.4 variants, including those identified here. We show that the strongest antibody responses in adults exposed to GII.4 Sydney 2012 are directed to previously circulating GII.4 viruses. Our data suggest that the broadening of antibody responses compromises establishment of strong GII.4 Sydney 2012 immunity, thereby allowing the continued persistence of GII.4 Sydney 2012 and modulating the cycle of norovirus GII.4 variant replacement. Our results indicate a cycle of norovirus GII.4 variant replacement dependent upon population immunity. Young children are susceptible to divergent variants; therefore, emergence of these strains worldwide is driven proximally by changes in adult serological immunity and distally by viral evolution that confers fitness in the context of immunity.

## INTRODUCTION

Human norovirus is the causative agent of ~20% of all acute gastroenteritis episodes worldwide (1). Although all age groups are infected, the most significant impact of disease is among young children and the elderly, who together bear the highest mortality rates, estimated at more than 200,000 deaths/year (2). This global disease burden has motivated the development of norovirus vaccines, with two candidates currently in clinical trials (3–5). Vaccine development is hampered by extensive diversity within the >35 known norovirus genotypes that infect humans (6). Recent progress in identifying key correlates of protection such as histo-blood group antigen (HBGA)-blocking antibodies (Ab) and the development of cell culture systems for several norovirus strains has accelerated vaccine development. Antigenic diversity within the GII.4 genotype, serotype/epitope-level cross-immunity, lack of understanding of the drivers of strain emergence, and the absence of a robust small animal model remain significant hurdles. Of these, antigenic diversity within the GII.4 genotype is the most significant obstacle to vaccine development (7–9). Norovirus global resurgence events occur cyclically and correspond with emergence of novel GII.4 variants characterized by unique constellations of residues within neutralizing antibody epitopes encoded within the capsid protein (VP1) (7, 9–11). Virus recombination between the genes that encode the RNA-dependent RNA polymerase (ORF1-NS7) and viral capsid (ORF2) types may also contribute to variant circulation and persistence in human populations (12–14).

Phylotemporal analysis of norovirus sequences suggests that preemergent GII.4 viruses with novel epitopes critical to immune escape circulate at endemic levels before becoming dominant, specifically in young children with limited GII.4 cross-protective immunity (15–17). Prior to global spread, these preemergent viruses would appear on phylogenetic trees at the end of atypically long branches. Population immunity would then determine which variants would successfully emerge and become dominant. As the most frequent cause of viral acute gastroenteritis outbreaks in the 21st century, six GII.4 norovirus variants have predominated worldwide in the past 25 years (18–20), resulting in significant burdens on health care, education, and military units (21–23). The first GII.4 variant with worldwide distribution emerged in the mid-1990s, followed by subsequent variant replacements in 2002 (circulation 2002 to 2005), 2004 (2004 to 2008), 2006 (2006 to 2011), 2009 (2009 to 2012), and 2012 (2012 to at least 2022). Notably, GII.4 Sydney 2012 viruses have not yet been replaced and continue to circulate globally and account for ~50% of norovirus outbreaks 10 years postemergence (24). Why this variant has persisted in the population for a decade is a fundamental question the field has yet to answer.

Here, based on preemergence diversification, we identified two candidate viruses fitting the phylogenetic characteristics of preepidemic variants and evaluated their emergence potential by comparing their susceptibilities to neutralization by sera from children and adults. Sera from young children were largely variant specific across time, whereas neutralization potency of sera from adults was broader with evidence of back-boosting to previously dominant GII.4 variants. These data support the idea that Sydney 2012 persistence may be driven by a lack of durable Sydney 2012-specific protective immunity in adults. Further, we found that continued exposure to GII.4 Sydney 2012 and antigenically related viruses results in a broadening of the immune response to include viruses characterized as potentially preemergent by phylogenetic analyses, potentially limiting new variant emergence. Consequently, we propose a model in which emerging variant selection is likely dependent upon the dynamics of the serological repertoire of adult populations with preexisting immunity, shaped and then reshaped by sequential GII.4 exposures (15, 25, 26).

## RESULTS

### Identification of highly divergent GII.4 variants by capsid gene sequence.

**FIG 1:**
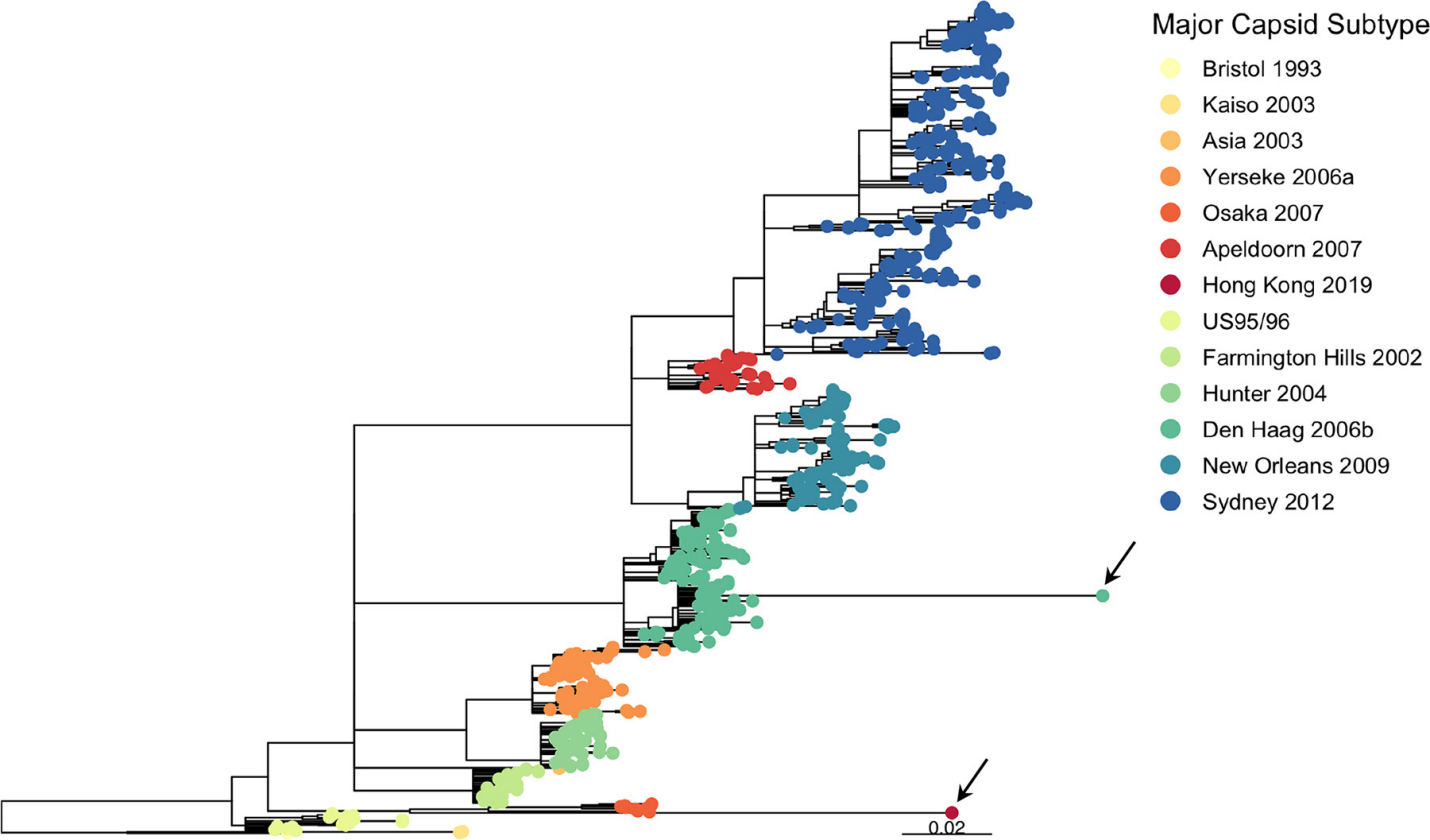
Maximum likelihood tree of 930 GII.4 major capsids generated by RAxML (66). Nodes with bootstrap support of less than 70 were collapsed. Rooting was optimized by TempEst (67). Tip color indicates major capsid subtype with epidemics in red tones and GII.4 viruses that spread globally in blue tones. Two long branches are observed: one leading to Hong Kong 2019 detected on 18 March 2019 and one leading to Den Haag 2017 detected on 21 February 2017. The corresponding tips are indicated by an arrow.

To search for viruses with preemergent signatures, we analyzed 930 GII.4 norovirus ORF2 sequences from samples collected in the United Kingdom between 1994 and 2019; all possessed full complementary metadata (including age, gender, geography, and date of sample). Two taxa with atypically long branches were observed in the phylogenetic tree (Fig. 1; see also Fig. S1 in the supplemental material). The first, GII.4 Hong Kong 2019, demonstrated a rate of evolution in accordance with the remainder of the data (Fig. S2). It is most closely related to the GII.4 Osaka 2007 variant, although it diverged from this lineage well before the Osaka 2007 epidemic, in keeping with characteristics observed in known preepidemic variants (15) (Fig. S3). The second, GII.4 Den Haag 2017, emerged from within the GII.4 Den Haag 2006 cluster (Fig. S3).

10.1128/mbio.01861-22.1 FIG S1 Distribution of internal and terminal branch lengths of phylogenetic tree of the major capsid nucleotide sequence of GII.4 variants. *x* axis, branch lengths—these indicate the number of substitutions per site, such that larger branch lengths correspond to larger genetic change having occurred. *y* axis, frequency, number of internal and terminal branches with corresponding branch length. The branch lengths of Den Haag 2017 and Hong Kong 2019 are the two largest in the data set of 930 GII.4 sequences. Download FIG S1, PDF file, 0.1 MB. Copyright © 2022 Lindesmith et al. 2022 Lindesmith et al. https://creativecommons.org/licenses/by/4.0/ This content is distributed under the terms of the Creative Commons Attribution 4.0 International license.

10.1128/mbio.01861-22.2 FIG S2 Root-to-tip divergence plot of major capsid nucleotide sequences of GII.4 variants. The significant linearity of the data suggests a clock-like signal in which all observed viruses are evolving at a similar rate. The *x* axis indicates isolation year, and the *y* axis shows the root-to-tip divergence on the maximum likelihood phylogenetic tree using TempEst. The black line indicates a linear regression line of the root-to-tip divergence and isolation year. A total of 930 sequences were included. Each variant is represented by a circle with Hong Kong 2019 and Den Haag 2017 colored according to the capsid subtype and all other sequences in gray (OTHER). Download FIG S2, PDF file, 0.1 MB. Copyright © 2022 Lindesmith et al. 2022 Lindesmith et al. https://creativecommons.org/licenses/by/4.0/ This content is distributed under the terms of the Creative Commons Attribution 4.0 International license.

10.1128/mbio.01861-22.3 FIG S3 Magnification of Den Haag 2017 and Hong Kong 2019 emergence from maximum likelihood tree of 930 GII.4 major capsids generated by RAxML (66) using 750 bootstraps shown in Fig. 1. Nodes with bootstrap support of less than 70 were collapsed. Rooting was optimized by TempEst (67). Tip color indicates major capsid subtype with endemic variants in red tones and epidemic variants in blue tones. Den Haag 2017, indicated by a black arrow, emerged from withing the Den Haag 2006 clade (left). Hong Kong 2019, indicated by a black arrow, shares a common ancestor with Osaka 2007 (right). Download FIG S3, PDF file, 0.1 MB. Copyright © 2022 Lindesmith et al. 2022 Lindesmith et al. https://creativecommons.org/licenses/by/4.0/ This content is distributed under the terms of the Creative Commons Attribution 4.0 International license.

**FIG 2:**
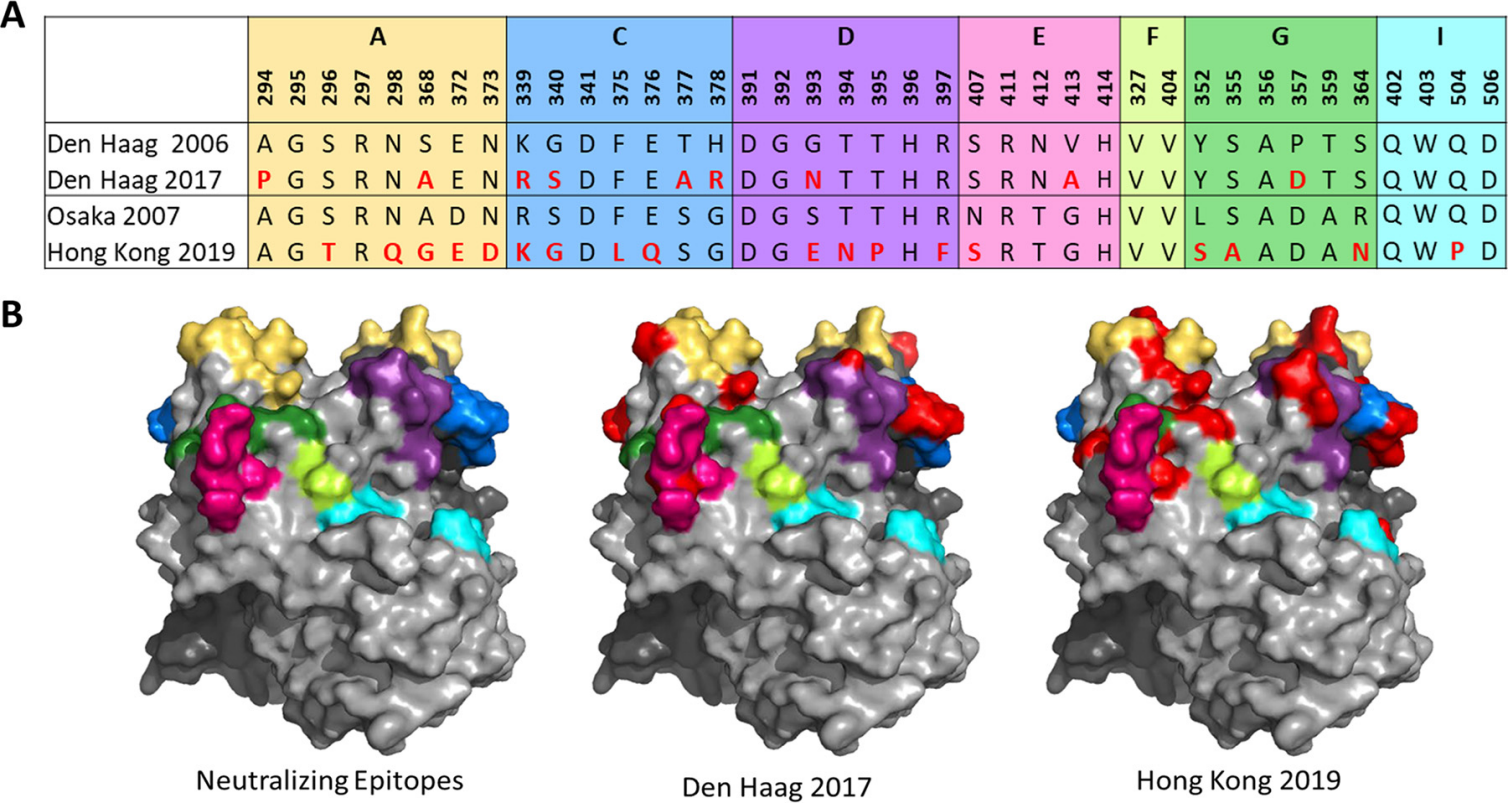
Sequence of GII.4 blockade/neutralizing antibody antigenic sites in divergent strains compared to those in closely related strains tested here. (A) Comparison of amino acid sequences in VP1 blockade/neutralizing antibody antigenic sites in Den Haag 2017 and Hong Kong 2019 to those in Den Haag 2006 and Osaka 2007 strains studied here as VLPs. (B) Blockade/neutralizing antibody antigenic sites mapped onto the surface of Sydney 2012 P domain dimer (PDB 4wzt). Residues differing between Den Haag 2017 and 2006 or Hong Kong 2019 and Osaka 2007 are colored red in both panels A and B. Complete capsid sequences for Den Haag 2019 and Hong Kong 2019 compared to their closest sequence neighbors are shown in Fig. S5.

Den Haag 2017 exhibits few changes in characterized antigenic regions (Fig. S4). It has 5 unique residues (294P, 339R, 340S, 378R, and 413A) relative to the 155 Den Haag 2006 strains in our data set; however, only 378R in antigenic site C is unique relative to all other strains in our data set. In contrast, Hong Kong 2019 possesses 16 unique antigenic site residues relative to the 10 Osaka 2007 strains in our data set (Fig. S4). Of these, six in antigenic sites A (298Q), D (393E, 395P, and 397F), and G (355A and 364N) are unique relative to all other strains in our data set. Unique combinations of residues in these sites are known to correlate with immune escape and widespread GII.4 variant emergence. Monitoring changes at known antigenic sites alone is insufficient to predict strain emergence. Indeed, multiple GII.4 variants with differences within known neutralizing/blockade Ab antigenic sites have been shown to cocirculate (15), yet only six became predominant and spread globally (18–20). Thus, we developed a phenotype-based testing pipeline to evaluate the impact of genotype changes. All GII.4 variants that have spread globally share two features: novel neutralizing/ligand binding blockade antibody antigenic site profiles and binding to multiple cellular ligands (8, 20, 27, 28). Human noroviruses use ABO histo-blood group antigens (HBGAs) as cellular binding ligands (29, 30). HBGA expression varies between and within human populations; thus, the ability to bind to a diverse set of ligands corresponds to a potentially large susceptible population. The HBGA ligand binding pocket that binds the common glycan among ABO antigens is highly conserved across GII.4 variants; however, variation within residues 391 to 397 (antigenic site D) provides stabilizing interactions with ligands that impact variant avidity for different ABO HBGAs as well as neutralizing antibodies (7, 8, 31–34). Den Haag 2017 and Hong Kong 2019 have substitutions within antigenic site D (Fig. 2). To investigate the phenotypic effect of these sequence changes on strain ligand binding, we developed representative virus-like particles (VLPs) for Den Haag 2017 and Hong Kong 2019 and matched ancestral strains of Den Haag 2006 and Osaka 2007 variants (Fig. 2).

10.1128/mbio.01861-22.4 FIG S4 Logoplot of antigenic sites across 930 sequences. (A) Den Haag 2017—unique residues relative to reference cluster (Den Haag 2006) and all other non-Den Haag 2017 sequences shown in blue and red boxes, respectively. (B) All Den Haag 2006. (C) All non-Den Haag 2017 sequences. (D) Hong Kong 2019—unique residues relative to reference cluster (Osaka 2007) and all other non-Hong Kong 2019 sequences shown in blue and red boxes, respectively. (E) All Osaka 2007. (F) All non-Hong Kong 2019 sequences. Download FIG S4, PDF file, 0.1 MB. Copyright © 2022 Lindesmith et al. 2022 Lindesmith et al. https://creativecommons.org/licenses/by/4.0/ This content is distributed under the terms of the Creative Commons Attribution 4.0 International license.

10.1128/mbio.01861-22.5 FIG S5 Capsid amino acid alignments of Den Haag 2006 compared to Den Haag 2017 (A) and Osaka 2007 (Armidale 2008) and Hong Kong 2019 (B). VP1 amino acid sequences were aligned in Geneious Prime 2021.2.2 using the Geneious Alignment tool with default settings. Residues that differ between the two sequences are shaded gray. Download FIG S5, PDF file, 0.1 MB. Copyright © 2022 Lindesmith et al. 2022 Lindesmith et al. https://creativecommons.org/licenses/by/4.0/ This content is distributed under the terms of the Creative Commons Attribution 4.0 International license.

### Evaluation of divergent variant emergence potential based on capsid ligand binding profiles.

**FIG 3:**
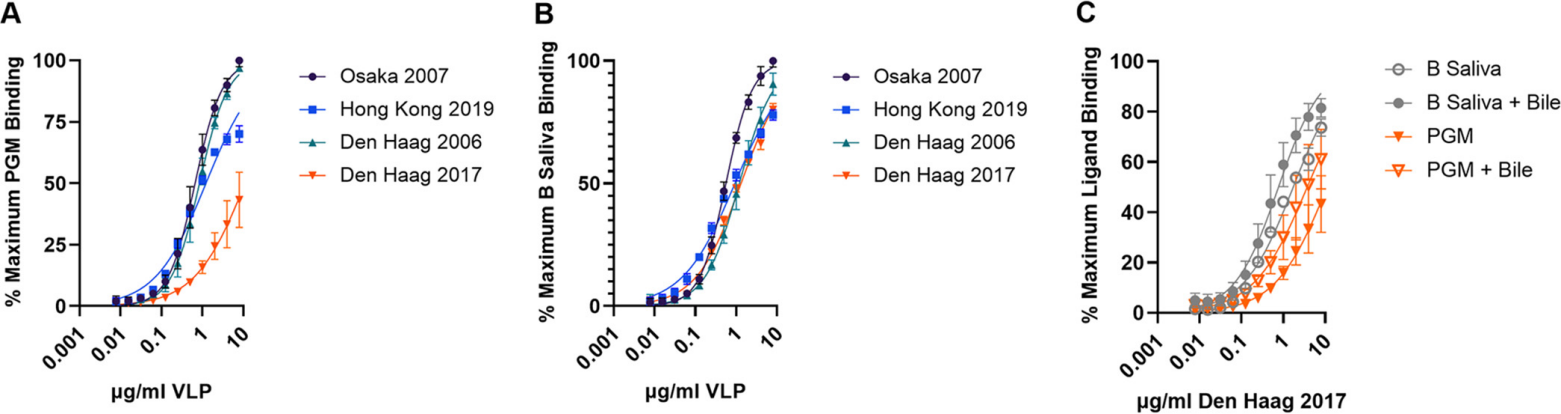
Impact of GII.4 variant evolution on ligand binding. GII.4 Osaka 2007, Hong Kong 2019, and Den Haag 2006 have typical GII.4 ligand binding patterns interacting with PGM (A) and B-type salivary ligands (B). Den Haag 2017 has lower avidity for PGM than but similar avidity for B-type saliva as the other GII.4 variants. Addition of bile stabilizes binding of Den Haag 2017 to ligands (C). Maximum binding was defined by Osaka 2007. Markers denote the mean and standard deviation from two replicates tested in two independent experiments.

Den Haag 2006, Osaka 2007, and Hong Kong 2019 displayed robust binding to native ligands in porcine gastric mucin (PGM) which includes H, A, and Lewis Y antigens and to human blood group B saliva (B antigen) (Fig. 3A to C and Fig. S6). Half-maximum binding titer (EC_50_) ranged from 0.66 to 1.2 μg/mL for PGM and 0.57 to 1.2 μg/mL for B saliva. Den Haag 2017 bound to B saliva as described for the other GII.4 VLPs (EC_50_, 1.4 μg/mL) but bound only weakly to PGM (EC_50_, >8 μg/mL). Den Haag 2017-PGM binding could be partially compensated by inclusion of bile (EC_50_, 3.7 μg/mL), a norovirus ligand binding and cellular infection cofactor (Fig. 3C and Fig. S6) (35–37). Retention of binding to B antigen is likely mediated by 393N as this residue has been previously shown to stabilize GII.4 binding to B antigen (8), although residues in antigenic site A may also contribute to HBGA binding affinity. No other tested GII.4 variants required bile for binding to PGM, indicating sufficient avidity for ligand interaction without cofactors (Fig. S6) (35). The dependence of Den Haag 2017 on bile for sufficient avidity potentially indicates that residue changes in Den Haag 2017 might be deleterious to viral fitness compared to other GII.4 variants. In contrast, Hong Kong 2019 interaction with cellular ligands was similar to that of other GII.4 variants (Fig. 3A and B). Inclusion of ligand binding characteristics suggests that the global outbreak potential of Den Haag 2017 viruses is relatively diminished while the potential for Hong Kong 2019 is maintained, based on avidity for the diverse sampling of cellular binding ligands found in the human population.

10.1128/mbio.01861-22.6 FIG S6 GII.4 variant ligand binding. (A to C) Mean optical density at 450 nm for each VLP binding to PGM or B saliva in the presence or absence of 1% bile was fit as single-site binding curves. Osaka 2007 (A), Hong Kong 2019 (B), and Den Haag 2006 (C) do not need bile for binding to PGM or B saliva. Den Haag 2017 has low avidity for PGM that is improved by addition of bile. (D) Markers denote the mean and standard deviation from two replicates tested in two independent experiments. Data in Fig. 3 represent a subset of the data presented here. Download FIG S6, TIF file, 0.5 MB. Copyright © 2022 Lindesmith et al. 2022 Lindesmith et al. https://creativecommons.org/licenses/by/4.0/ This content is distributed under the terms of the Creative Commons Attribution 4.0 International license.

### Evaluation of divergent variant emergence potential based on population serum antibody reactivity.

**FIG 4:**
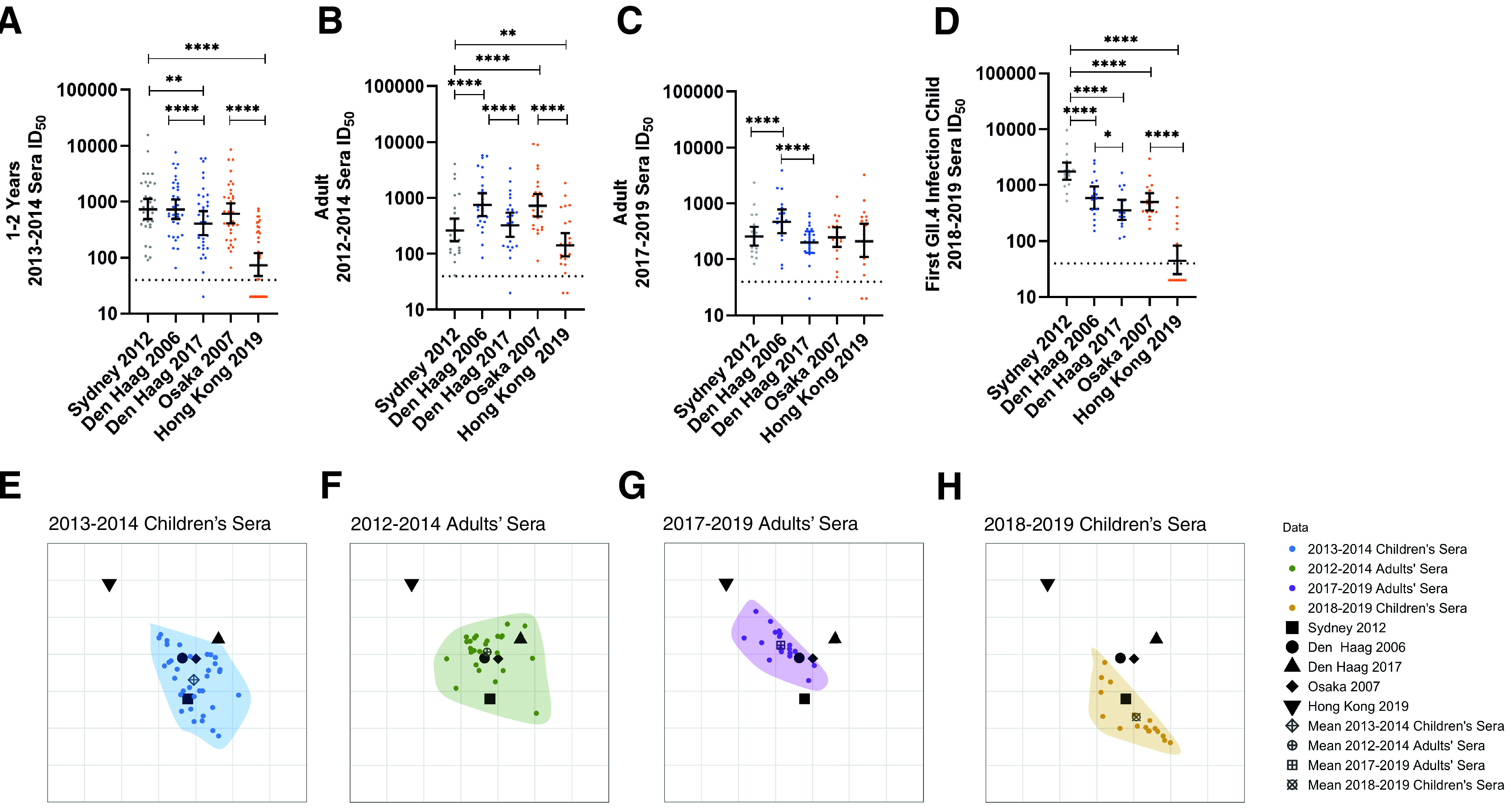
Antigenic divergence of novel strains depends on exposure history of the population. (A to D) Blockade antibody titer in sera from children aged 1 to 2 years in 2013 to 2014 (*n* = 34) (A), adults in 2012 to 2014 (*n* = 25) (B), adults in 2017 to 2019 (*n* = 19) (C), and children (*n* = 17) after their first GII.4 (Sydney 2012) infection in 2018 to 2019 (D). Marker, individual response. Line, geometric mean titer. Error bars, 95% confidence intervals. *, *P* ≤ 0.03; **, *P* ≤ 0.0021; ****, *P* < 0.0001, Wilcoxon matched-pairs signed-rank test, compared to Sydney 2012 or between the two Den Haag VLPs or Osaka 2007 and Hong Kong 2019. (E to H) Antigenic cartography analysis across all serum sets, divided into separate panels for clarity: children in 2013 to 2014 (E), adults in 2012 to 2014 (F), adults in 2017 to 2019 (G), and children in 2018 to 2019 (H). Virus variants are represented as distinct black shapes, and each serological data set is represented by distinct colors and with data points encapsulated in a shaded polygon. The mean of the serological points for each serum set is indicated by distinct dark gray shapes. One grid box corresponds to a 2-fold change in titer. Note that the orientation of the plots does not matter.

Among individuals genetically susceptible to GII.4 infection (those who express ABO antigens on mucosal surfaces), human norovirus GII.4 global outbreak potential depends on the presence of key residues in neutralizing Ab (NAb) antigenic sites that allow the virus to escape prevailing population immunity (7, 8, 25, 27, 38, 39). Here, we evaluated the antigenicity of Den Haag 2017 and Hong Kong 2019 in the context of prevalent population immunity at the peak of Sydney 2012 circulation in the United Kingdom in 2013 to 2014 (40) (Fig. 4), by measuring the potency of blockade of ligand binding in a surrogate neutralization assay. Sera were collected from young children (1 to 2 years) and healthy adults (≥15 years) in 2013 to 2014 and in 2012 to 2014, respectively (41). Individual infection history is unknown, but norovirus is the leading cause of gastroenteritis in the United Kingdom (42). To exclude samples from individuals potentially genetically resistant to GII.4 infection, only sera with a GII.4 Sydney blockade antibody titer of ≥40 were included in the analyses (young children, *n* = 34; healthy adults, *n* = 25). Although cross-sectional samples are unable to capture changes in an individual’s response over time, population patterns of responses may be observed for sets of sera.

Among children sampled in 2013 to 2014, the blockade potencies for Den Haag 2006, Osaka 2007, and Sydney 2012 were similar (geometric mean titer [GMT] range, 679 to 811) (Fig. 4A), even though these children had likely been predominantly, although not exclusively, exposed to Sydney 2012, based on child age and year of sample collection. In comparison, the blockade potencies against the newly observed Den Haag 2017 and Hong Kong 2019 variants by children’s sera were substantially lower, by 2- and 9-fold, respectively, than those against Sydney 2012. Despite being collected at the height of Sydney 2012 predominance, the adult sera from 2012 to 2014 had the strongest blockade potency against Den Haag 2006 (GMT, 825) and Osaka 2007 (GMT, 798), with Sydney 2012 lower by 3-fold (Fig. 4B). The blockade of Den Haag 2017 was 2-fold lower, similar to that observed with the children’s sera, while blockade of Hong Kong 2019 was 5-fold lower.

To evaluate these findings in the context of more contemporary population immunity, we made the same comparison with sera collected from healthy adults in 2017 to 2019 (*n* = 19) (Fig. 4C) and young children with a confirmed first GII.4 infection sequence verified as Sydney 2012 (median age, 12 months [interquartile range {IQR}, 7 to 13 months], *n* = 17) (Fig. 4D). As shown for adult sera in 2012 to 2014, blockade antibody potency among adults in 2017 to 2019 was skewed toward Den Haag 2006, 2-fold higher than that against Sydney 2012. In addition, blockade titers were not statistically significantly different between Sydney 2012, Den Haag 2017, Osaka 2007, and Hong Kong 2019 (GMT range, 214 to 271; Wilcoxon *P* > 0.05). Titer to Den Haag 2006 was not statistically significantly different between the 2012–2014 and 2017–2019 adult serum sets (Mann-Whitney *P* = 0.23). In comparison, titers in sera collected from children recently experiencing their first GII.4 infection with Sydney 2012 in 2018 to 2019 were highly skewed toward Sydney 2012 (GMT, 1,810) with decreased titers to Den Haag 2006 (3-fold), Den Haag 2017 (5-fold), Osaka 2007 (4-fold), and Hong Kong 2019 (35-fold). These data support back-boosting of antibody responses to previous GII.4 variants after Sydney 2012 infection in adults with previous GII.4 exposure.

### The breadth of antigenic divergence of the novel variants depends upon the exposure history of the population.

To better understand and quantify the antigenic relationships between the GII.4 variants over time and between sets of sera, we used antigenic cartography to visualize the antigen distance (AD) of the viruses to each other compared to each serum set (43–45). One AD equals a 2-fold change in blockade antibody response. A 4-fold change is associated with GII.4 norovirus immune escape in immunized mice (9, 46). We performed a combined analysis including all four sets of sera and all 5 variants. Figure 4E to H shows the antigenic relationships where we have illustrated each serum set separately for visual clarity. The serum sets are shown together in Fig. S7A. Representation of the data in two dimensions (2D) recapitulated the distance matrix generated from 50% inhibitory dilution (ID_50_) values as well as the three-dimensional (3D) mapping (Fig. S8A, *R*^2^ = 0.70 and 0.73, respectively). All antigenic distances are reported to the nearest integer. For clarity, we discuss the mean AD for each serum set relative to the variants.

**FIG 5:**
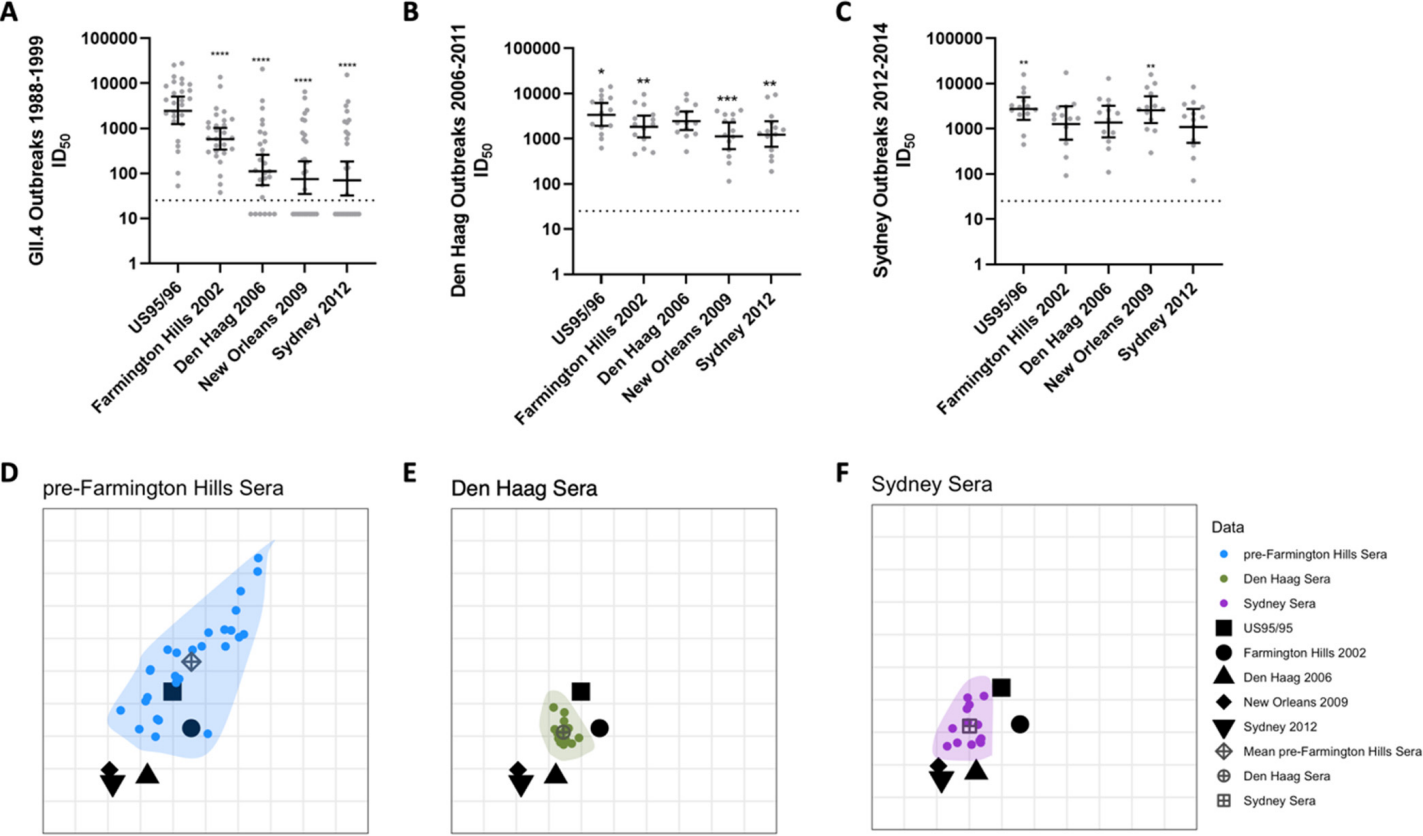
Antigenic divergence measured by convalescent-phase outbreak sera. (A to C) Blockade antibody titer in adult sera collected from GII.4 norovirus outbreaks between 1988 and 1999 (*n* = 27) (A), Den Haag 2006 outbreaks in 2006 to 2011 (*n* = 14) (B), and Sydney outbreaks in 2012 to 2014 (*n* = 14) (C) were compared to time-ordered GII.4 variants. Marker, individual response. Line, geometric mean titer. Error bars, 95% confidence intervals. *, *P* ≤ 0.03; **, *P* ≤ 0.0021; ***, *P* ≤ 0.0002; ****, *P* < 0.0001, Wilcoxon matched-pairs signed-rank test, compared to US95/96 (A), Den Haag 2006 (B), or Sydney 2012 (C). (D to F) Antigenic cartography analysis across all serum sets, divided into separate panels for clarity: pre-Farmington Hills sera (D), Den Haag 2006 sera (E), or Sydney 2012 sera (F). Viral variants are represented as distinct black shapes, and each serological data set is represented by distinct colors and is encapsulated in a shaded polygon. The mean of the serological points for each serum set is indicated by distinct dark gray shapes. One grid box corresponds to a 2-fold change in titer. Note that the orientation of the plots does not matter.

10.1128/mbio.01861-22.7 FIG S7 Two-dimensional antigenic cartography of GII.4 noroviruses. (A) All panels shown in Fig. 4E and F. (B) All panels shown in Fig. 5D to F. Viral variants are represented as distinct black shapes, and each serological data set is represented by distinct colors and is encapsulated in a shaded polygon. The mean of the serological points for each serum set is indicated by distinct dark gray shapes. Note that the orientation of the plots does not matter. One antigenic distance (one grid box) corresponds to a 2-fold change in titer. Download FIG S7, PDF file, 0.1 MB. Copyright © 2022 Lindesmith et al. 2022 Lindesmith et al. https://creativecommons.org/licenses/by/4.0/ This content is distributed under the terms of the Creative Commons Attribution 4.0 International license.

10.1128/mbio.01861-22.8 FIG S8 Two-dimensional and three-dimensional antigenic cartography of GII.4 noroviruses recapitulate normalized titers equally well. (A) Antigenic distance, dij, versus normalized titers, Dij, for 2D (left) and 3D (right) cartography derived from surveillance sera in Fig. 4. (B) Antigenic distance, dij, versus normalized titers, Dij, for 2D (left) and 3D (right) cartography derived from outbreak sera in Fig. 5. *R*^2^ scores from the linear regression models are shown inset on the respective plots. Download FIG S8, PDF file, 0.3 MB. Copyright © 2022 Lindesmith et al. 2022 Lindesmith et al. https://creativecommons.org/licenses/by/4.0/ This content is distributed under the terms of the Creative Commons Attribution 4.0 International license.

The sera collected from children in 2018 to 2019 clusters most closely with Sydney 2012, with an antigenic distance of 1 between the mean for the sera and Sydney 2012. This is consistent with Sydney 2012 being the first and only GII.4 exposure among these children. In contrast, the sera collected from children in 2013 to 2014 are distributed around Sydney 2012, Den Haag 2006, and Osaka 2007 and close to Den Haag 2017 (all ADs to mean for the sera are 1), indicating children with multiple GII.4 infections develop blockade antibody responses with more breadth than those of singly GII.4-infected children. A similar trend is observed in sera collected from adults in 2012 to 2014, where the sera are clustered around Den Haag 2006 and Osaka 2007, which are closest to the mean (AD to mean for sera of 0), with substantial overlap with Den Haag 2017 and Sydney 2012, which are equidistant to the mean (AD to the mean for sera of 1). Only the adult sera collected in 2017 to 2019 extend toward all norovirus GII.4 variants, including Hong Kong 2019. While the mean for the adult sera collected in 2017 to 2019 still retains its proximity to Den Haag 2006 and Osaka 2007 variants (AD to the mean for sera of 1), the distance of the mean to the Hong Kong 2019 variant is equal to the distance from Sydney 2012 and Den Haag 2017 (AD to the mean for sera of 2), indicating repeat Sydney exposure may be broadening the GII.4 blockade antibody response overall rather than eliciting Sydney-specific responses.

Together, these findings suggest that in naive individuals, antibodies to Sydney 2012 are highly cross-reactive with the antigenically similar Den Haag 2006 and Osaka 2007, while individuals previously exposed to GII.4 norovirus variants show evidence of Den Haag 2006 and Osaka 2007 boosting. Further exposure to Sydney 2012 in previously GII.4-exposed individuals seems to extend existing cross-reactivity to include divergent new variants, even when there is little likelihood of the individuals having ever been exposed to these new variants. In contrast, primary GII.4 infection sera do not result in broad cross-reactivity and are therefore less likely to protect against divergent new variants.

### Repeat GII.4 exposure broadens antibody immunity.

Of the four sets of sera compared above, only the children’s sera collected between 2018 and 2019 have a known GII.4 norovirus infection history (Sydney 2012 infection within the previous 7 months), and these sera have antibody responses specific to the infecting variant. In the other sets of sera comprising individuals with evidence of previous immunity to GII.4 viruses, it is possible that antibody responses specific to a new infecting variant waned faster than the responses of preexisting antibodies, now boosted by reinfection. This could explain the broad antibody responses we see in these populations. To evaluate this, we compared the blockade antibody titer of convalescent-phase sera collected ~21 days after GII.4 infection to those of a panel of time-ordered GII.4 variants spanning from the first known norovirus variant that spread globally in the mid-1990s (US95/96), through to Sydney 2012 (Fig. 5 and Table S1). Convalescent-phase sera after GII.4 infection between 1988 and 1999 preferentially targeted US95/96 (GMT, 2920) with moderate cross-reactivity to Farmington Hills 2002 (4-fold decrease in GMT) but poor cross-reactivity to Den Haag 2006 (18-fold decrease) and New Orleans 2009 and Sydney 2012 (both 24-fold decrease) (Fig. 5A), indicating infection with the first globally dominant US95/96 GII.4 variant, or related viruses, did not result in broad antibody responses at the peak of titer, in agreement with observations on immunity after primary infection with Sydney 2012.

10.1128/mbio.01861-22.9 TABLE S1 GII.4 outbreak demographics. Download Table S1, PDF file, 0.1 MB. Copyright © 2022 Lindesmith et al. 2022 Lindesmith et al. https://creativecommons.org/licenses/by/4.0/ This content is distributed under the terms of the Creative Commons Attribution 4.0 International license.

In contrast, convalescent-phase sera after Den Haag 2006 and Sydney 2012 infection were broadly potent across the time-ordered panel of variants with the GMT varying between 0.7- and 2-fold compared to the Den Haag 2006 or Sydney 2012 GMT (Fig. 5B and C). Den Haag serum blockade antibody titers were most potent for US95/96 (GMT of 3,562) followed by Den Haag 2006 (GMT of 2,557), with reduced cross-reactivity to Farmington Hills 2002 (GMT of 1,939), New Orleans 2009 (GMT of 1,227), and Sydney 2012 (GMT of 1,339). Serum titers after Sydney 2012 infection were highest to US95/96 (GMT of 2,899) and New Orleans 2009 (GMT of 2,762) with titers similar to those to Sydney 2012 (GMT of 1,247), Farmington Hills 2002 (GMT of 1,445), and Den Haag 2006 (GMT of 1,536). Thus, convalescent-phase sera collected at the peak of antibody titer indicate that the broad antibody responses characterized in sera from individuals with previous GII.4 exposures are not the result of rapid waning of antibody to the current infecting variant, as individuals infected with Den Haag 2006 or Sydney 2012 also produced maximum titers to noninfecting variants. In all three sets of outbreak sera, GII.4 infection predominantly back-boosted antibodies to US95/96, even when the infecting strain was significantly different. Further, in none of the sets of sera (surveillance or GII.4 outbreaks) likely to have experienced multiple GII.4 infections based on age and variant circulation was the antibody response to Sydney 2012 infection driven predominantly by anti-Sydney 2012 specific antibodies.

As with the surveillance sera, we employed antigenic cartography to better understand and quantify the antigenic relationships between the viral variants relative to the three outbreak sera in 2D (Fig. 5D to F; Fig. S7B). 2D representation recapitulated the distance matrix generated from ID_50_ values as well as, if not better than, the 3D mapping (Fig. S8B; both *R*^2^ = 0.65 and 0.60, respectively).

The variants cluster into two distinct groups: US95/96 and Farmington Hills 2002, and Den Haag 2006, New Orleans 2009, and Sydney 2012. The antigenic distance between the two clusters (AD range of 2 to 3) is larger than that within each group (AD range of 0 to 1). The antigenic dissimilarity between these two clusters is in line with the shift in antigenicity that has previously been observed in human sera to coincide with the emergence of the Den Haag 2006 variant (7, 11).

Convalescent-phase sera before Farmington Hills infection cover the largest antigenic space, potentially reflecting the large span of time of sample collection (1988 to 1999), the mean for which is closest to the US95/96 and Farmington Hills 2002 variants (AD of 1 and 2, respectively). In contrast, convalescent-phase sera after Den Haag 2006 infection occupy a much smaller antigenic space, perhaps reflecting the shorter time frame of observation (2006 to 2011). The corresponding mean for these sera is equidistant and closest to strains preceding and including Den Haag 2006 (US95/96, Farmington Hills 2002, Den Haag 2006, AD of 1) and slightly further away from later dominant variants (New Orleans 2009 and Sydney 2012, AD of 2). Similar to convalescent-phase sera after Den Haag 2006 infection, sera after Sydney 2012 infection exhibit a compact distribution. The distribution of these sera retains its proximity to Den Haag 2006 (AD to mean for sera of 1) while broadening in the direction of the other variants (AD to mean of 2) in agreement with trends of back-boosting and broadening of GII.4 antibody responses identified in surveillance sera.

Taken together with the results from nonoutbreak sera, these data illustrate the complexities of population responses, suggesting that population immunity to human norovirus broadens by exposure and is shaped by preexposure histories with back-boosting of previous immunity.

## DISCUSSION

Many human norovirus GII.4 variants cocirculate globally without causing increased levels of disease. Global emergence and global predominance of GII.4 noroviruses likely require use of a broad selection of binding ligands with change in a combination of viral characteristics (novel neutralizing epitopes) and host factors (susceptible genetic background and ineffective population immunity). By applying the metrics described here for evaluating the potential for immune evasion and broad host range to viruses selected for sequence divergence, we have established a potential pipeline for predicting the potential of GII.4 human noroviruses to spread globally. Similar approaches may also be applicable to other acute viral infections with cocirculating variants that lack tractable laboratory systems for functional analyses (such as *in vitro* culture). Although both Den Haag 2017 and Hong Kong 2019 have unique residue changes in antigenic sites, the limited number of changes of Den Haag 2017 compared to other Den Haag 2006 strains, its emergence from within the Den Haag 2006 cluster, and its weak interaction with cellular ligands may hinder the potential for Den Haag 2017 to escape from population immunity and efficient transmission. While these data suggest that Hong Kong 2019 is better poised to infect a broad population, to emerge as a dominant variant Hong Kong 2019 would need to evade current population immunity. This variant is able to evade antibody in adult and child sera from 2013 to 2014 and child sera from 2018 to 2019. In contrast, adult sera from 2017 to 2019 contain effective blocking antibodies. This may explain why, although Hong Kong 2019-like strains have occasionally been detected in Asia and Europe, including the United Kingdom, they have failed to increase in numbers. Den Haag 2017 is more effectively blocked by all sera, which may explain why we find no evidence of any similar sequences among our own collection or in any public database.

Understanding the host and virologic factors that facilitate the transition of a GII.4 variant from causing low-level disease in children/asymptomatic infection in adults to a variant that spreads globally, causing symptomatic disease in adults, would increase the reliability of predicting which variants will spread globally and inform vaccine development. GII.4 variants cocirculate and evolve for years, specifically in children (15–17), and it is host factors that appear to be the major driving force that discriminates between variants that cause endemic disease versus those that spread globally. Adults likely become susceptible to symptomatic infection when either antibody neutralization titers wane at mucosal sites or infection reshapes the serological repertoire of circulating neutralizing/blockade antibodies and provides an avenue for the emergence of a preexisting, epitope-divergent variant with good fitness. Thus, GII.4 variant threat prioritization lists will vary over time and depend on immunity in adults with extensive preexposure history and back-boosted memory antibody responses to human norovirus.

Analyzing sera from exposed and recently infected children and adults demonstrates the complex immune profile that develops from single and sequential exposures to norovirus GII.4 variants. Following the first GII.4 infection, blockade antibody responses demonstrated more specificity to the infecting variant with limited cross-reactivity to other variants, even closely related variants. Subsequent GII.4 variant infection, as has likely occurred in the children and adults sampled in 2013 to 2014, broadened the antibody response to closely related variants (Den Haag 2006 and Osaka 2007) but not the more distant variant (Hong Kong 2019). Finally, repeat exposure to the Sydney 2012 variant over the past decade, based on known variant prevalence data, further broadened the antibody response to include more distant variants such as Hong Kong 2019 in adults sampled in 2017 to 2019. This antibody broadening was replicated within an additional set of commercial adult sera collected in 2019 and correlated with targeting of antigenic site G (41). In adults with a history of exposure to prior GII.4 variants, immunological back-boosting resulted in a mismatch between the current variant and the resulting immune response. For both adult surveillance serum data sets, blockade antibody titers to the Den Haag 2006 variant are higher than those to the Sydney 2012 variant. Taken together, these data suggest that exposure to Sydney 2012 over time results in a broadening of the immune response rather than a recalibrating of the immune response to preferentially neutralize Sydney 2012. Alternatively, there may be key residue changes in 2012 Sydney that induce highly cross-reactive antibody responses, potentially through conformational changes as been described for HIV (47, 48), influenza virus (49), and norovirus and SARS-CoV-2 (50). This widening of immune breadth, and subsequent narrowing of available antigenic space, may explain the lack of a novel dominant variant to yet replace Sydney 2012. Subsequently, waning of population immunity resulting either in lower titer or in reshaping of the serological repertoire by selective loss of specific antibodies, as may have occurred during COVID-19-related behavior modifications, may allow a new variant to emerge.

Supporting these conclusions, using antigenic cartography to compare the relationships between each of the population serum sets and viruses indicates that the breadth of viruses that are neutralized changes over time. Adult sera from 2012 to 2014 and 2017 to 2019 cluster most closely with the Den Haag 2006 and Osaka 2007 viruses, despite continued exposure to Sydney 2012 in the latter group. However, the breadth of viruses neutralized by adult sera from 2017 to 2019 is greater, as evidenced by the proximity of the sera and all variants analyzed. In contrast, child sera from 2012 to 2013 and from 2018 to 2019 both clustered closer to Sydney 2012, the likely most recent infecting strain. However, child sera from 2012 to 2013, with potentially multiple different GII.4 exposures, were more evenly distributed across the variants than child sera from 2018 to 2019 following a single primary exposure. Similar patterns of antigenic breadth were observed with outbreak sera soon after known GII.4 variant infection. Taken together, one interpretation is that the antibody response to Sydney 2012 in adults drives strong GII.4 memory B cell responses to Den Haag 2006. Strong memory B cell responses to Den Haag 2006 could, in turn, reduce antibody responses to Sydney 2012, reflected in the comparably longer mean antigenic distances. This hypothesis is in line with previous findings that GII.4 human norovirus antibody responses in adults postvaccination are primarily memory responses to ancestral GII.4 variants (26, 51). Moreover, human monoclonal antibodies and human polyclonal sera indicate Den Haag 2006 and Sydney 2012 (as well as New Orleans 2009) are antigenically more similar to each other than to previous variants (7, 28, 52). Notably, independent serological and cartographic analyses of GII.4 outbreak sera with known exposure history verify the pattern of broadening immunity and back boosting described in surveillance sera, suggesting that the above observations do not result from selective waning of antibody responses postinfection but instead reflect the complex dynamics of changing population immunity to human norovirus.

Young children should be the primary target for norovirus vaccination if the aim is to prevent infection and decrease burden of disease (53–55). To be most effective, infants with limited GII.4 exposure will need to be immunized. Here, we show that sera from children are less cross-reactive than sera from adults, particularly following a first GII.4 infection. This finding fits with data showing that younger infants and single-variant-immunized mice have been shown to respond primarily to the infecting variant (9, 46, 56, 57). Greater breadth of immunity likely develops over time as a consequence of multiple infections. How the order or frequency of these infections impacts antibody responses and subsequently serologic and mucosal immunity is unknown but will affect the cross-protection patterns and vaccine formulation requirements as new variants emerge and population immunity shifts. The correlation between serological and mucosal immunity for norovirus and the impact of each on protection against infection and disease is not well studied, especially considering the disease burden.

Incorporating population immunity into a conceptual model of GII.4 variant global emergence likely improves the accuracy of the results but also has challenges. Accurate prediction will require the establishment of a large panel of reagents for the testing pipeline, which is especially complex within the context of continually evolving population immunity and necessitates a continued requirement for contemporary human samples or complex immunization schemes for animals. Here, we assembled a unique resource of sera comprised of both year-matched archived and year-matched contemporary sera. However, we were unable to match the serum sets demographically. Specifically, the first GII.4 infection child sera were collected in a low- to middle-income country with less genetic diversity than the source of the other sera, the United Kingdom and United States. While these features likely impact the likelihood of GII.4 exposure or infection for a child, they are unlikely to directly explain the differences in serum breadth discussed here, as only individuals with serological evidence of GII.4 infection (genetically susceptible) were included in the analyses and the first infection breadth of blockade antibody responses in the children is similar to those reported after single GII.4 immunization of animals and those seen in adults after ancestral GII.4 infection (8, 27).

It also remains uncertain for how long variants might circulate before emergence. It is likely that regular and repeat modeling of adult and child sera will be necessary to differentiate between antibody cross-reactivity and preexposure with defined molecular diagnostics techniques. The roles that adult parents of young children play in accelerating or mitigating transitions of new emergent variants to global outbreaks will also need to be defined. Further, we cannot exclude at this time the possibility that the increase in the numbers of children in the population who are susceptible to a particular GII.4 variant (e.g., Hong Kong 2019), relative to adults with immunity, may itself increase the chance of a global spread of a variant. How these factors interplay with the fact that children initially exposed to Sydney 2012 will have different antibody set points for back-boosting and cross-reactivity has yet to be determined but will guide both the spread of new GII.4 variants and vaccine component requirements in the future. Several of these issues could be addressed by implementing national surveillance systems not only for outbreaks but also for sporadic norovirus cases as well as environmental samples that may better reflect the diversity of infection. These questions apply not only to human norovirus but also to other RNA viruses under selective pressure.

The physical isolation brought on by the COVID-19 pandemic has decreased hospitalizations for influenza, respiratory syncytial virus (RSV), rotavirus, and norovirus infections (58). Norovirus reports decreased by an estimated 40 to 79% during the COVID-19 pandemic (59–61). These stark declines support the potential effectiveness of nonpharmaceutical interventions at controlling norovirus transmission and emphasize the impact that effective surveillance and control measures (e.g., alert systems linked to public health messaging) could have in the absence of a licensed vaccine. Fewer human norovirus infections during the COVID-19 pandemic imply that population-wide antibody titers have likely waned, potentially setting the stage for emergence of a new GII.4 variant (62).

## MATERIALS AND METHODS

### Variant data set.

Nine hundred thirty GII.4 norovirus samples, with full metadata, collected and sequenced as part of the NoroPatrol Surveillance Project were analyzed here.

### Fecal sample collection.

Fecal specimens from norovirus RNA-positive patients were referred from outbreak events detected by local and regional National Health Service (NHS) and Public Health England (PHE) laboratories to the National Enteric Virus Reference Laboratory at PHE Colindale. All samples were confirmed norovirus RNA positive by real-time reverse transcription-PCR (RT-PCR) (63) and genotyped by partial capsid sequencing, targeting either region C (64) or the hypervariable P2 domain (65) in open reading frame 2 (ORF2) of the norovirus genome, in accordance with local standard operating procedures.

Fecal specimens were prepared as 10% (wt/vol) suspensions in balanced salt solution (minimal essential medium; Life Technologies), and viral RNA was purified from the supernatants of clarified suspensions using the QIAsymphony, operating the Complex200_v4 protocol with the DSP virus/pathogen kit (Qiagen), eluting RNA in a final volume of 60 μL. Quantity of norovirus RNA in each sample was estimated by real-time RT-PCR to establish a cycle threshold (*C_T_*) value, and samples with a *C_T_* value of <35 were selected for whole-genome sequencing.

### Library preparation and whole-genome sequencing.

SuperScript IV (Life Technologies) was used to synthesize single-stranded cDNA from RNA samples. This was followed immediately by second-strand synthesis using the NEBNext mRNA second-strand synthesis module (New England Biolabs) to generate double-stranded cDNA which was purified using AMPure XP magnetic beads (Beckman Coulter). Libraries were prepared using the SureSelect XT low-input reagent kit (Agilent). Briefly, the cDNA was sheared into fragments of ~300 bp using the E220 focused ultrasonicator (Covaris), after which ends were repaired, adenosine tails were added, and adapters were ligated. Adapter-ligated libraries were amplified, incorporating a unique index into each library to allow multiplexed pooling. Libraries were then hybridized with custom-designed biotinylated norovirus RNA baits, and baits were captured using streptavidin-coated magnetic beads. After a second amplification step, the final libraries were quantified, pooled in equimolar amounts, and sequenced on a MiSeq sequencer (Illumina) using a V2 500-cycle kit for 250-bp paired-end reads.

### Mapping.

Raw read data in paired-end Fastq files were first adapter trimmed, followed by removal of low-quality (<Q30) reads. The reads that passed quality control were then mapped to a large custom data set of norovirus genotype references. From the multimapping BAM file we selected the best reference for the data set based on the number of reads mapping to it and the completeness of the genome. In cases of mixed infection, multiple top references were selected. The reads were then remapped to the best reference or the best set of references (in cases of mixed infection), duplicate reads were removed, and then consensus sequence was called at a minimum depth of 10 reads. Complete analysis was carried out using CLC Genomics Workbench v 11.01. The consensus sequences (Fasta format) are then genotyped using the norovirus genotyping tool hosted at https://www.rivm.nl/mpf/typingtool/norovirus/.

### Phylogenetic analysis.

The maximum likelihood tree of the alignment was constructed using RAxML (66) with the GTR model and 750 bootstrap replicates. The temporal signal of the data set was explored using TempEst (67). The optimum tree root was identified by maximizing the R squared correlation coefficient. Trees were visualized using ggtree (68–70). VP1 amino acid sequences of Den Haag 2006/Den Haag 2017 and Osaka 2007/Hong Kong 2019 were aligned in Geneious Prime 2021.2.2 using the Geneious Alignment tool with default settings.

### Serum samples.

Sera collected in the United Kingdom were obtained from two sources: sera from children 1 to 2 and 15 years of age were obtained from the Public Health England Seroepidemiology Unit (PHE SEU), and sera from adults collected in the 2012–2014 FluWatch were obtained from the Health Survey for England Biobank (41). The use of coded serum samples was approved by the National Health Service Research Ethics Committee (reference 17/EE/0269), London School of Hygiene and Tropical Medicine (reference LEO12196), University of North Carolina at Chapel Hill (18-0214), and CDC (IRB no. 5051). Sera collected in 2017 to 2019 from adults were purchased from BioIVT (Hicksville, NY). Sera were obtained from a birth cohort study in León, Nicaragua, following the subjects’ first reverse transcription-quantitative PCR (RT-qPCR)-confirmed symptomatic Sydney 2012 infection. The study was approved by the Institutional Review Boards of the National Autonomous University of Nicaragua, León (UNAN-León, Acta Number 45, 2017) and the University of North Carolina at Chapel Hill (study number 16-2079). Children experiencing their first GII.4 infection were a median of 12 months old (IQR, 7 to 13 months), and the convalescent-phase sera were collected less than 7 months after GII.4 infection. Outbreak sera were selected from GII.4 outbreaks between 1988 and 2014 based on year and norovirus strain. Convalescent-phase sera were selected from norovirus RT-qPCR-confirmed cases. The epidemiological and demographic data available are summarized in Table S1 in the supplemental material. All sera were received coded with no link back to donor identification and were heat inactivated for 30 min at 56°C before use.

### Structural homology modeling.

Blockade/neutralizing antibody antigenic sites were mapped onto the surface of Sydney 2012 P domain dimer (PDB 4wzt) and with the PyMOL Molecular Graphics System, version 2.0 (Schrödinger, LLC).

### VLP production.

Virus-like-particle (VLP)-matched strain accession numbers are in Table S2.

10.1128/mbio.01861-22.10 TABLE S2 GenBank accession numbers for VLPs used in this study. Download Table S2, PDF file, 0.07 MB. Copyright © 2022 Lindesmith et al. 2022 Lindesmith et al. https://creativecommons.org/licenses/by/4.0/ This content is distributed under the terms of the Creative Commons Attribution 4.0 International license.

ORF2 genes of all strains except Sydney 2012 (MZ376651) were synthesized by Bio Basic Inc. (Amherst, NY) and inserted directly into the Venezuelan equine encephalitis virus replicon vector for production of VLPs in baby hamster kidney cells (ATCC CCL-10) as described previously (71, 72). Sydney 2012 (MZ376651) VLPs were produced from baculovirus vectors as described previously (10). VLP particle integrity was verified by ligand and antibody binding and visualization of ~40-nm particles by electron microscopy.

### VLP-ligand binding assays.

VLPs bound to pig gastric mucin type III (PGM; 10 μg/mL) (Sigma-Aldrich) or human type B saliva were detected by rabbit polyclonal antiserum (Cocalico Biologicals, Stevens, PA), as described previously (39, 73). If included, 1% bovine bile salts (Sigma-Aldrich) were diluted in phosphate-buffered saline (PBS), aliquoted, and stored at −20°C until use (35, 36).

### Blockade of VLP-ligand binding assays.

Ligand binding blockade antibody assays were performed as described previously (73, 74). VLPs were pretreated with decreasing concentrations of sera for 1 h and then transferred to ligand-coated plates for 1 h, and bound VLPs were detected as for ligand binding assays. PGM (10 μg/mL) was the ligand source for the Den Haag 2006, Osaka 2007, and Hong Kong 2019 assays. B saliva supplemented with 1% bile was the ligand source for the Den Haag 2017 assay (35, 36). The percent control binding was compared to that for no serum pretreatment. Mean 50% inhibitory dilution (ID_50_) titers and 95% confidence intervals (95% CIs) were determined from log(inhibitor) versus normalized response-variable slope curve fit in GraphPad Prism 9.1.2 (26, 51). Sera that did not block at least 50% of VLP binding to ligand at the lowest dilution tested were assigned a titer equal to 0.5 times the lowest tested dilution for statistical analysis.

### Antigenic cartography.

All ID_50_ titer measurements were used in the process of antigenic cartography. The antigenic map in 2D and 3D was made with Racmac software implemented in RStudio, which implements the methodology as in reference 43. Shaded polygons are defined using function geom_encircle (https://CRAN.R-project.org/package=ggalt). The data visualizations were created using tidyverse, ggplot2, ggalt, and ggforce packages (https://CRAN.R-project.org/package=ggalt, https://ggplot2.tidyverse.org/).

### Statistical analysis.

Blockade antibody titer and ligand binding statistical analyses were performed using GraphPad Prism 9.1.2 (7, 26). ID_50_ values were determined by nonlinear curve fit of normalized data with variable slope (7, 26). ID_50_ values were log transformed for analysis and compared by Wilcoxon matched-pairs signed-rank test when comparing VLPs for a serum set or by Mann-Whitney test when comparing serum sets for the same VLP. Ligand binding data were fit with log(inhibitor) versus response-variable slope (four parameters) for EC_50_ calculation or maximum binding and dissociation constants by single-site binding curve analysis in GraphPad Prism 9.1.2 (75). A difference was considered significant if *P* was <0.05.

### Data availability.

The sequence of Den Haag 2017 and Hong Kong 2019 are available on GenBank with accession codes OK376714.1 and MT742777.1 respectively. ID50 values, coordinates in antigenic space and code used to generate figures are available on GitHub (https://github.com/ftettamanti/norovirus-antigenic-cartography).
