# Supplementary figures and images for: Immune Imprinting Drives Human Norovirus Potential for Global Spread
Source: mBio. 2022 Sep 14;13(5):e01861-22. doi: 10.1128/mbio.01861-22 (PMC9600701; doi:10.1128/mbio.01861-22)

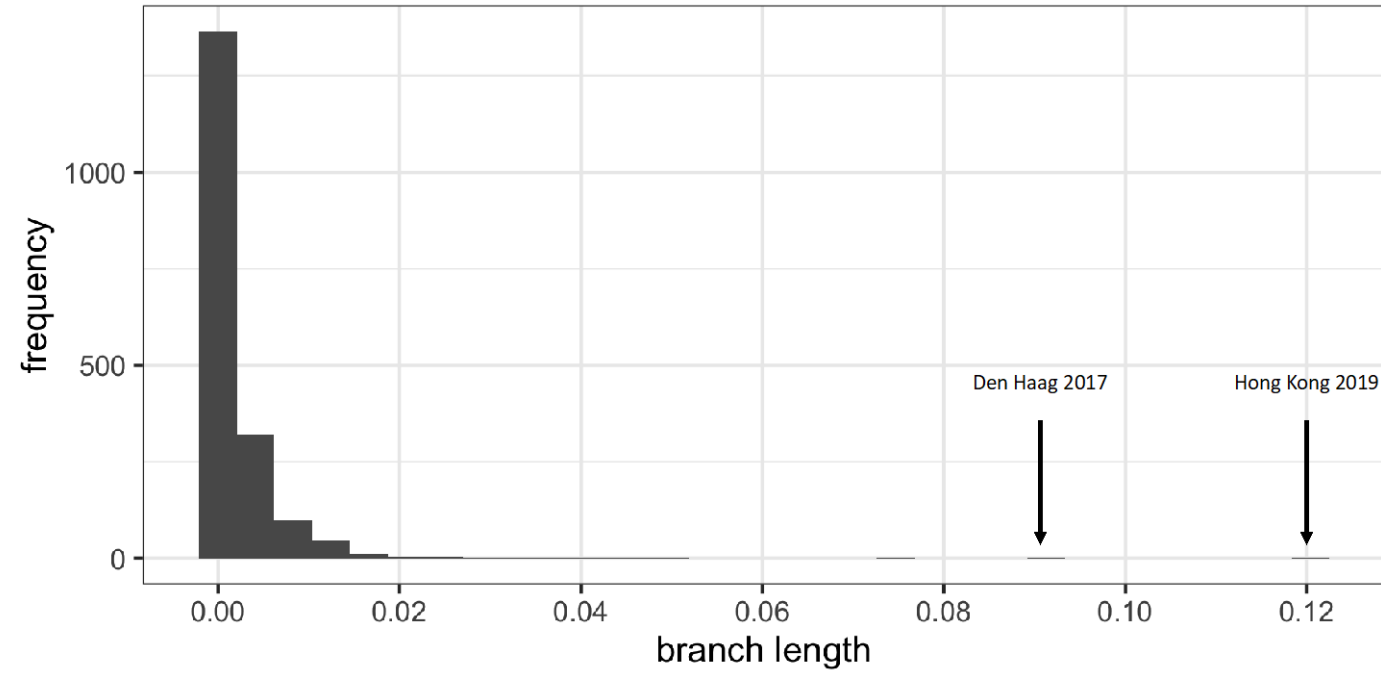

Supplement: FIG S1 [file mbio.01861-22-s0001.pdf]

Root-to-tip GII.4 Capsid

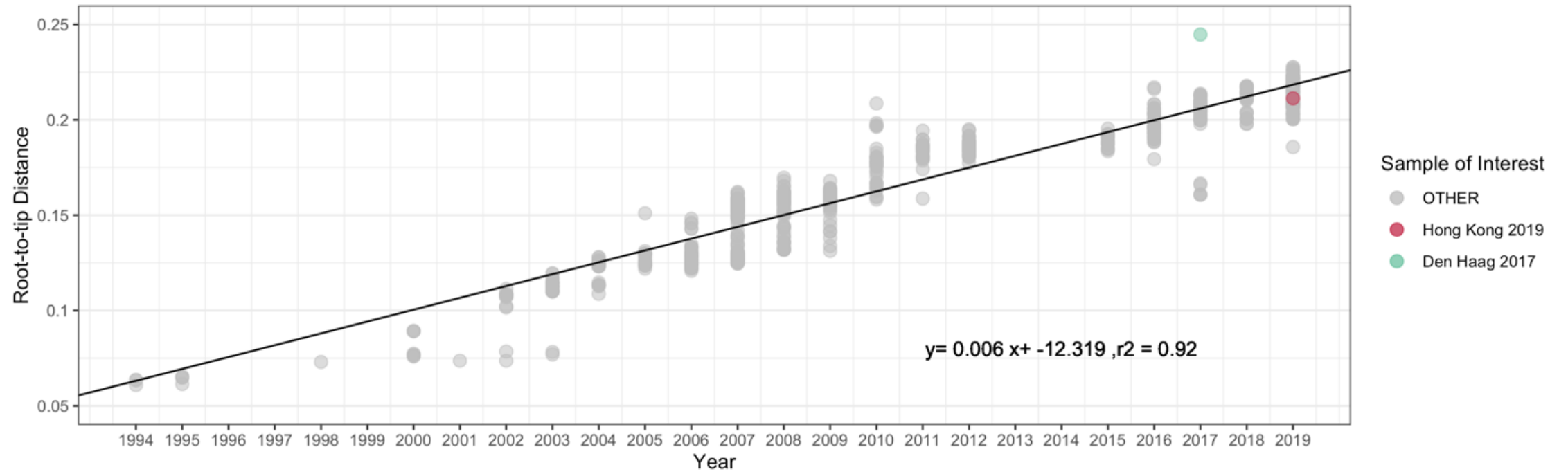

Supplement: FIG S2 [file mbio.01861-22-s0002.pdf]

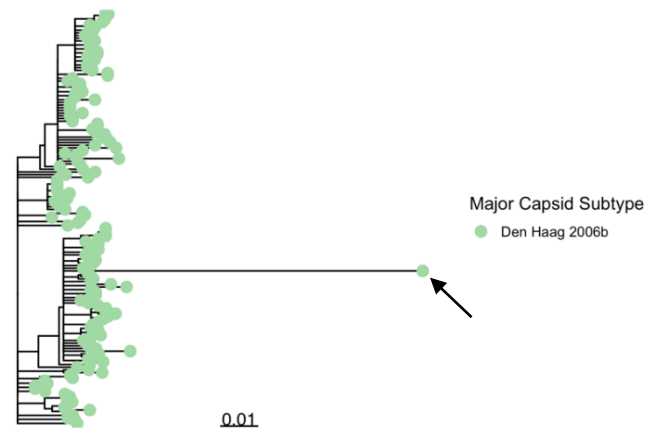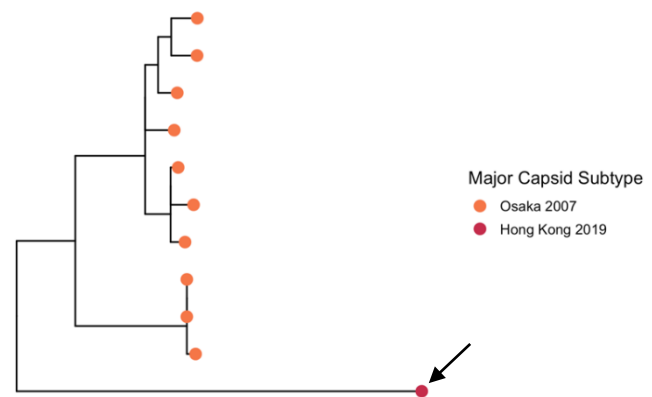

Supplement: FIG S3 [file mbio.01861-22-s0003.pdf]

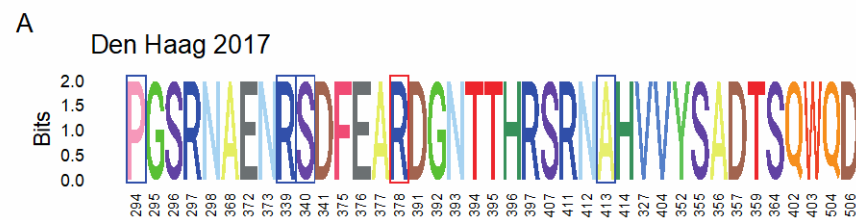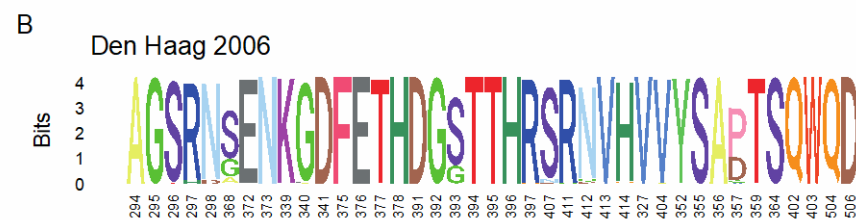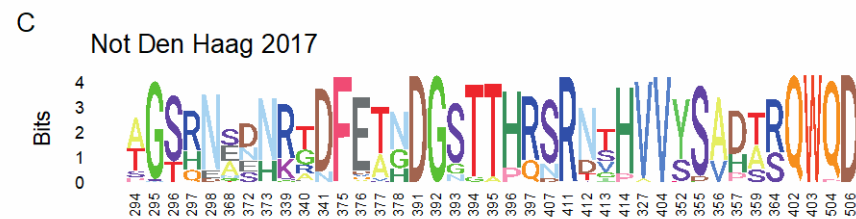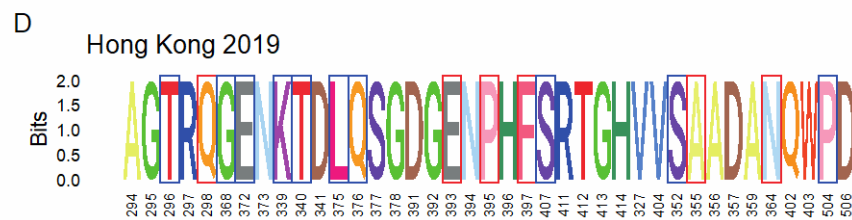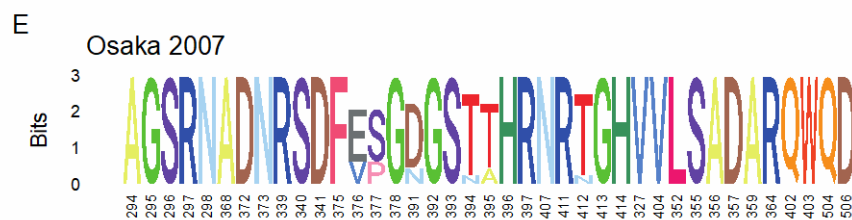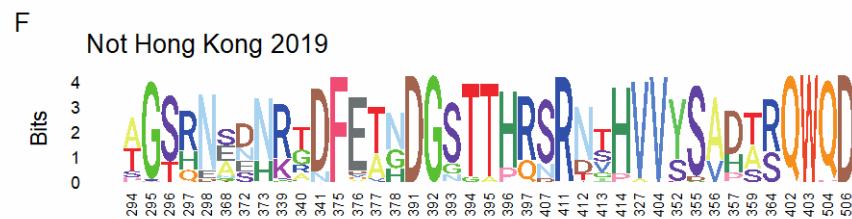

Supplement: FIG S4 [file mbio.01861-22-s0004.pdf]

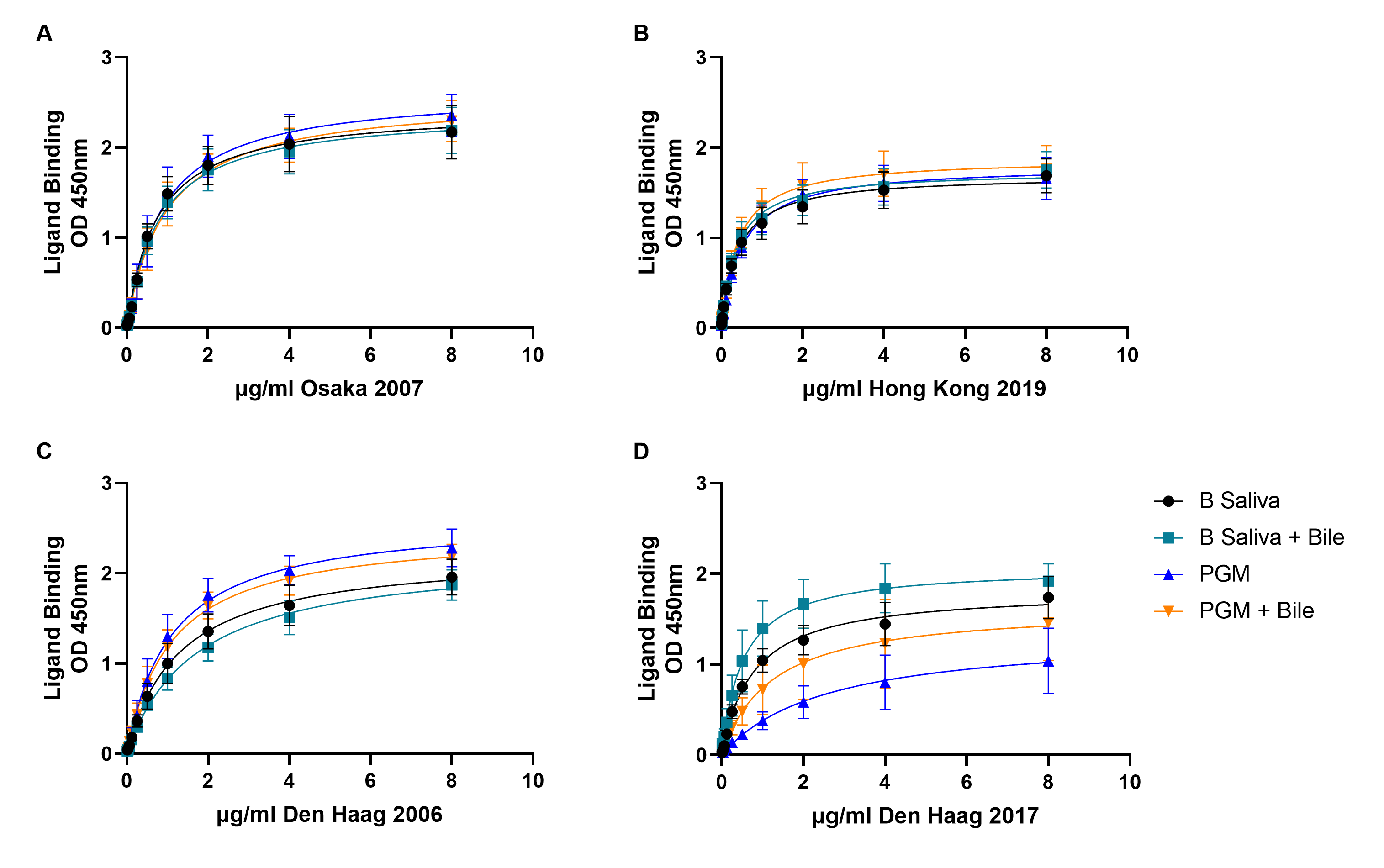

Supplement: FIG S6 [file mbio.01861-22-s0006.tif]

**A**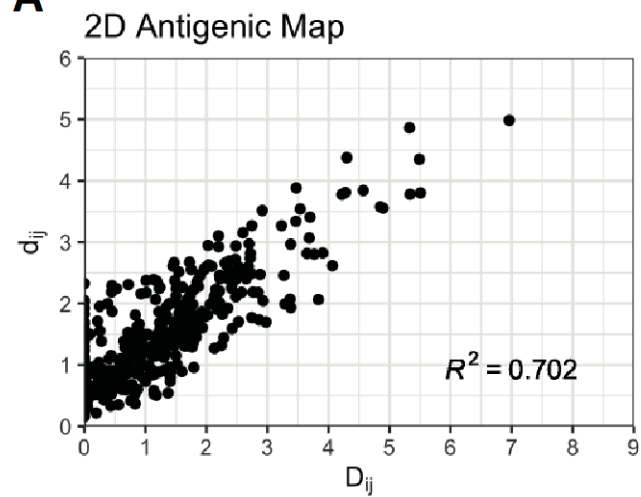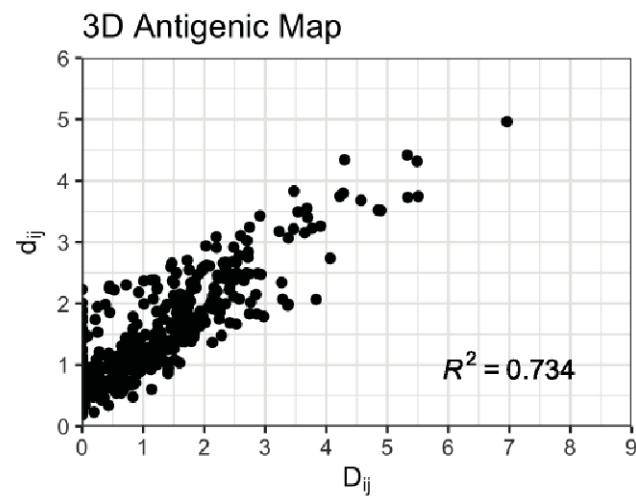**B**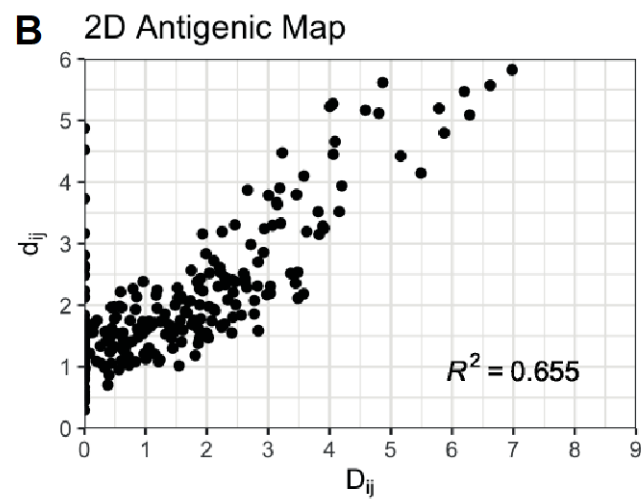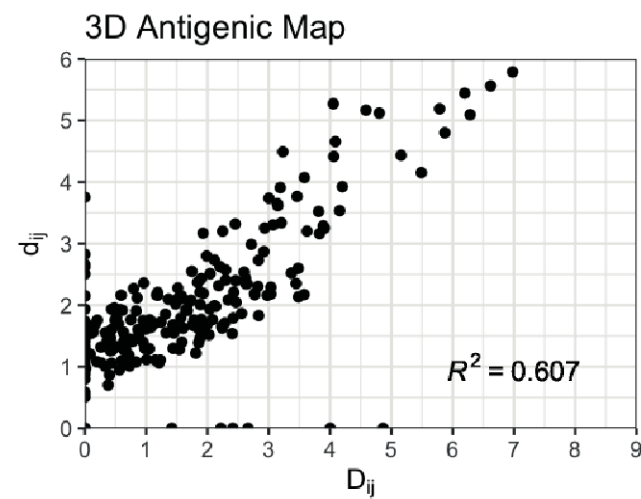

Supplement: FIG S8 [file mbio.01861-22-s0008.pdf]
