# Supplementary material for: Immune Imprinting Drives Human Norovirus Potential for Global Spread
Source: mBio. 2022 Sep 14;13(5):e01861-22. doi: 10.1128/mbio.01861-22 (PMC9600701; doi:10.1128/mbio.01861-22)
Supplement: FIG S5 [file mbio.01861-22-s0005.pdf]

A.

```
                         10        20        30        40        50        60        70        80        90        100
Consensus       MKMASNDANPSDGSAANLVPXVNNEVMALEPVXGAAIAAPVAGQQNVIDPWIRNNFVQAPGGEFTVSPRNAPGEILWSAPLGPDLNPYLSHLARMYNGYA  100
Den Haag 2006   MKMASNDANPSDGSAANLVPEVNNEVMALEPVVGAAIAAPVAGQQNVIDPWIRNNFVQAPGGEFTVSPRNAPGEILWSAPLGPDLNPYLSHLARMYNGYA  100
Den Haag 2017   MKMASNDANPSDGSAANLVPGVNNEVMALEPVAGAAIAAPVAGQQNVIDPWIRNNFVQAPGGEFTVSPRNAPGEILWSAPLGPDLNPYLSHLARMYNGYA  100

                         110       120       130       140       150       160       170       180       190       200
Consensus       GGFEVQVILAGNAFTAGKIIFAAVPPNFPTEGLXPSQVTMFPHXIXDVRQLEPVLIPLPDVRNNFYHYNQSNDSTJKLIAMLYTPLRANNAGXDVFTVSC  200
Den Haag 2006   GGFEVQVILAGNAFTAGKIIFAAVPPNFPTEGLSPSQVTMFPHIIVDVRQLEPVLIPLPDVRNNFYHYNQSNDSTIKLIAMLYTPLRANNAGEDVFTVSC  200
Den Haag 2017   GGFEVQVILAGNAFTAGKIIFAAVPPNFPTEGLNPSQVTMFPHVITDVRQLEPVLIPLPDVRNNFYHYNQSNDSTLKLIAMLYTPLRANNAGDDVFTVSC  200

                         210       220       230       240       250       260       270       280       290       300
Consensus       RVLTRPSPDFDFIFLVPPTVESXTKPFTXPILTVEEMTNSRFPIPLEKLFTGPSXAFVVQPQNGRCTTDGVLLGTTQLSPVNICTFRGDVXHIXGSRNYT  300
Den Haag 2006   RVLTRPSPDFDFIFLVPPTVESRTKPFTVPILTVEEMTNSRFPIPLEKLFTGPSGAFVVQPQNGRCTTDGVLLGTTQLSPVNICTFRGDVTHIAGSRNYT  300
Den Haag 2017   RVLTRPSPDFDFIFLVPPTVESKTKPFTIPILTVEEMTNSRFPIPLEKLFTGPSSAFVVQPQNGRCTTDGVLLGTTQLSPVNICTFRGDVAHIPGSRNYT  300

                         310       320       330       340       350       360       370       380       390       400
Consensus       MNLASLNWXNYDPTEEIPAPLGTPDFVGKIQGVLTQTTXXDGSTRGHKATVYTGSAXFTPKLGSVQXXTDTENDFEXXQNXKFTPXGVIQDGXTTHRNEP  400
Den Haag 2006   MNLASLNWNNYDPTEEIPAPLGTPDFVGKIQGVLTQTTKGDGSTRGHKATVYTGSAPFTPKLGSVQFSTDTENDFETHQNTKFTPVGVIQDGGTTHRNEP  400
Den Haag 2017   MNLASLNWSNYDPTEEIPAPLGTPDFVGKIQGVLTQTTRSDGSTRGHKATVYTGSADFTPKLGSVQLATDTENDFEARQNAKFTPIGVIQDGNTTHRNEP  400

                         410       420       430       440       450       460       470       480       490       500
Consensus       QQWVLPSYSGRNXHNVHLAPAVAPXFPGEQLLFFRSTXPGCSGYPNXDLDCLLPQEWVQHFYQZAAPAQSDVALLRFVNPDTXRVLFECKLHKXGYXTVX  500
Den Haag 2006   QQWVLPSYSGRNVHNVHLAPAVAPTFPGEQLLFFRSTMPGCSGYPNMDLDCLLPQEWVQHFYQEAAPAQSDVALLRFVNPDTGRVLFECKLHKSGYVTVA  500
Den Haag 2017   QQWVLPSYSGRNAHNVHLAPAVAPSFPGEQLLFFRSTLPGCSGYPNLDLDCLLPQEWVQHFYQQAAPAQSDVALLRFVNPDTSRVLFECKLHKAGYLTVS  500

                         510       520       530       540
Consensus       HTGQHDLVIPPNGYFRFDSWVNQFYTLAPMGNGTGRRRAL*                                                             541
Den Haag 2006   HTGQHDLVIPPNGYFRFDSWVNQFYTLAPMGNGTGRRRAL*                                                             541
Den Haag 2017   HTGQHDLVIPPNGYFRFDSWVNQFYTLAPMGNGTGRRRAL*                                                             541
```

B.

```
                         10        20        30        40        50        60        70        80        90        100
Consensus       MKMASNDANPSDGSTANLVPEVNNEVMALEPVVGAAIAAPVAGQQNXIDPWIRNNFVQAPGGEFTVSPRNAPGEILWSAPLGPDLNPYLSHLARMYNGYA  100
Hong Kong 2019  MKMASNDANPSDGSTANLVPEVNNEVMALEPVVGAAIAAPVAGQQNLIDPWIRNNFVQAPGGEFTVSPRNAPGEILWSAPLGPDLNPYLSHLARMYNGYA  100
Armidale 2008   MKMASNDANPSDGSTANLVPEVNNEVMALEPVVGAAIAAPVAGQQNVIDPWIRNNFVQAPGGEFTVSPRNAPGEILWSAPLGPDLNPYLSHLARMYNGYA  100

                         110       120       130       140       150       160       170       180       190       200
Consensus       GGFEVQVILAGNAFTAGKIIFAAVPPNFPTEGLSPSQVTMFPHXIVDVRQLEPVLIPLPDVRNNFYHYNQSNDXTIKLIAMLYTPLRANNAGDDVFTVSC  200
Hong Kong 2019  GGFEVQVILAGNAFTAGKIIFAAVPPNFPTEGLSPSQVTMFPHVIVDVRQLEPVLIPLPDVRNNFYHYNQSNDSTIKLIAMLYTPLRANNAGDDVFTVSC  200
Armidale 2008   GGFEVQVILAGNAFTAGKIIFAAVPPNFPTEGLSPSQVTMFPHIIVDVRQLEPVLIPLPDVRNNFYHYNQSNDPTIKLIAMLYTPLRANNAGDDVFTVSC  200

                         210       220       230       240       250       260       270       280       290       300
Consensus       RVLTRPSPDFDFIFLVPPTVESRTKPFTVPILTVEEMTNSRFPIPLEXLYTGPSXAFVVQPQNGRCTTDGVLLGTTQLSAVNICTFRGDXTHIAGXRXXT  300
Hong Kong 2019  RVLTRPSPDFDFIFLVPPTVESRTKPFTVPILTVEEMTNSRFPIPLEKLYTGPSTAFVVQPQNGRCTTDGVLLGTTQLSAVNICTFRGDLTHIAGTRQFT  300
Armidale 2008   RVLTRPSPDFDFIFLVPPTVESRTKPFTVPILTVEEMTNSRFPIPLERLYTGPSSAFVVQPQNGRCTTDGVLLGTTQLSAVNICTFRGDVTHIAGSRNYT  300

                         310       320       330       340       350       360       370       380       390       400
Consensus       MXLASXNWNNYDPTEEIPAPLGTPDFVGKIQGMLTQTTXXDGSTRGHKAXVXTGXADFAPKXGXXXFXTDTXBDXZSGXNTKFTPXGVXQDGXXXHXXEP  400
Hong Kong 2019  MHLASPNWNNYDPTEEIPAPLGTPDFVGKIQGMLTQTTKGDGSTRGHKAIVSTGAADFAPKSGNIRFGTDTEDDLQSGTNTKFTPIGVVQDGENPHFSEP  400
Armidale 2008   MNLASQNWNNYDPTEEIPAPLGTPDFVGKIQGMLTQTTRSDGSTRGHKATVLTGSADFAPKLGRVQFATDTDNDFESGQNTKFTPVGVIQDGSTTHRNEP  400

                         410       420       430       440       450       460       470       480       490       500
Consensus       QQWVLPXYXGRTGHNVHLAPAVAPTYPGEQLLFFRSTMPGCSGYPNXDJDCLLPQEWVQHFYQEAAPAQSDVALLRFVNPDTGRVLFECKLHKSGYVTVA  500
Hong Kong 2019  QQWVLPSYTGRTGHNVHLAPAVAPTYPGEQLLFFRSTMPGCSGYPNLDIDCLLPQEWVQHFYQEAAPAQSDVALLRFVNPDTGRVLFECKLHKSGYVTVA  500
Armidale 2008   QQWVLPNYSGRTGHNVHLAPAVAPTYPGEQLLFFRSTMPGCSGYPNMDLDCLLPQEWVQHFYQEAAPAQSDVALLRFVNPDTGRVLFECKLHKSGYVTVA  500

                         510       520       530       540
Consensus       HTGXHDLXIPPNGYFRFDSWVNQFYTLAPMGNGTGRRRXL*                                                             541
Hong Kong 2019  HTGPHDLAIPPNGYFRFDSWVNQFYTLAPMGNGTGRRRVL*                                                             541
Armidale 2008   HTGQHDLVIPPNGYFRFDSWVNQFYTLAPMGNGTGRRRAL*                                                             541
```
