# Supplementary material for: Immune Imprinting Drives Human Norovirus Potential for Global Spread
Source: mBio. 2022 Sep 14;13(5):e01861-22. doi: 10.1128/mbio.01861-22 (PMC9600701; doi:10.1128/mbio.01861-22)
Supplement: FIG S7 [file mbio.01861-22-s0007.pdf]

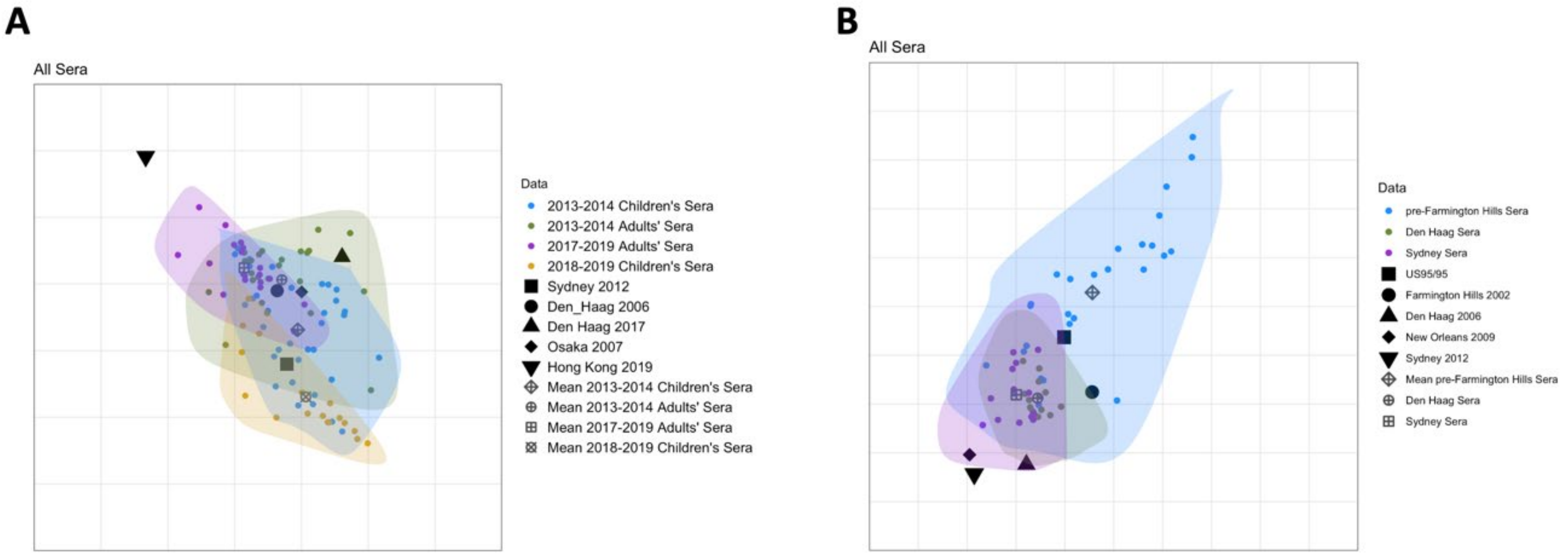
A
All Sera
Data
2013-2014 Children's Sera
2013-2014 Adults' Sera
2017-2019 Adults' Sera
2018-2019 Children's Sera
Sydney 2012
Den_Haag 2006
Den Haag 2017
Osaka 2007
Hong Kong 2019
Mean 2013-2014 Children's Sera
Mean 2013-2014 Adults' Sera
Mean 2017-2019 Adults' Sera
Mean 2018-2019 Children's Sera
B
All Sera
Data
pre-Farmington Hills Sera
Den Haag Sera
Sydney Sera
US95/95
Farmington Hills 2002
Den Haag 2006
New Orleans 2009
Sydney 2012
Mean pre-Farmington Hills Sera
Den Haag Sera
Sydney Sera
