# Supplementary material for: Immune Imprinting Drives Human Norovirus Potential for Global Spread
Source: mBio. 2022 Sep 14;13(5):e01861-22. doi: 10.1128/mbio.01861-22 (PMC9600701; doi:10.1128/mbio.01861-22)
Supplement: TABLE S1 [file mbio.01861-22-s0009.pdf]

Outbreak ID	Time MMM-YY	VP1 genotype	N° of cases	Participant's Age§		State	Source	Setting
				Average	Range			
1988-1	Apr-88	GII.4	51.0	66	24-93	SD	unknown	Long-term Care Facility
1988-2	Apr-88	GII.4	n/a	31	16-44	MI	unknown	High School
1995-1	Sep-95	GII.4	n/a	89.2	83-92	HI	unknown	Long-term Care Facility
1996-1	Jan-96	GII.4	40.0	n/a	n/a	SC	Person to person	Hospital
1997-1	Mar-97	GII.4	41.0	81.5	79-84	PA	unknown	Long-term Care Facility
1998-1	Mar-98	GII.4	125.0	20	19-21	TX	food	University
1999-1	Dec-99	GII.4	55.0	n/a	n/a	WV	Person to person	Long-term Care Facility
2006-1	Oct-06	GII.4 Den Haag	n/a	72.75	63-84	MS	Person to person	Cruise ship
2010-1	May-2010	GII.4 Den Haag	76.0	77	73-81	OR	Person to person	Long-term Care Facility
2011-1	Apr-2011	GII.4 Den Haag	25.0	53.5	27-80	OR	Person to person	Long-term Care Facility
2011-2	Sep-2011	GII.4 Den Haag	47.0	79	64-94	OR	Person to person	Long-term Care Facility
2012-1	Nov-2012	GII.4 Sydney	12.0	54.75	23-76	OR	Unknown	Long-term Care Facility
2012-2	Dec-2012	GII.4 Sydney	8.0	65	65	OR	unknown	Long-term Care Facility
2014-1	Mar-2014	GII.4 Sydney	n/a	45	45	OR	Person to person	Long-term Care Facility
2014-2	Mar-2014	GII.4 Sydney	n/a	49.4	23-76	OR	Person to person	Long-term Care Facility
2014-3	Apr-2014	GII.4 Sydney	19.0	37.3	25-46	OR	unknown	Long-term Care Facility

§ For those included in this study
