# Supplementary material for: Immune Imprinting Drives Human Norovirus Potential for Global Spread
Source: mBio. 2022 Sep 14;13(5):e01861-22. doi: 10.1128/mbio.01861-22 (PMC9600701; doi:10.1128/mbio.01861-22)
Supplement: TABLE S2 [file mbio.01861-22-s0010.pdf]

Variant	GenBank #
**Population Sera**	
Den Haag 2006	AFU92665.1
Den Haag 2017	UBX38815.1
Osaka 2007	ACX31885.1
Hong Kong 2019	QEL43936.1
Sydney 2012	AGJ52172.1
**Outbreak Sera**	
Camberwell 1987	AAK50355.1
GII.4 US 95/96	AFJ04707.1
Farmington Hills 2002	AFJ04708.1
Den Haag 2006	AFJ04709.1
Sydney 2012	AFV08795.1
